# Unravelling Alkali‐Metal‐Assisted Domain Distribution of Quasi‐2D Perovskites for Cascade Energy Transfer toward Efficient Blue Light‐Emitting Diodes

**DOI:** 10.1002/advs.202200393

**Published:** 2022-05-13

**Authors:** Wanqing Cai, Muhammad Umair Ali, Ping Liu, Miao He, Cong Zhao, Ziming Chen, Yue Zang, Man‐Chung Tang, Hong Meng, Hongyan Fu, Guodan Wei, Hin‐Lap Yip

**Affiliations:** ^1^ Tsinghua‐Berkeley Shenzhen Institute (TBSI) Tsinghua University Shenzhen 518055 China; ^2^ Tsinghua Shenzhen International Graduate School Tsinghua University Shenzhen City 518055 China; ^3^ State Key Laboratory of Luminescent Materials and Devices Institute of Polymer Optoelectronic Materials and Devices School of Materials Science and Engineering South China University of Technology 381 Wushan Road Guangzhou 510640 P. R. China; ^4^ School of Advanced Materials Peking University Shenzhen Graduate School Shenzhen City 518055 P. R. China; ^5^ Department of Materials Science and Engineering City University of Hong Kong Kowloon 999077 Hong Kong; ^6^ School of Energy and Environment City University of Hong Kong Kowloon 999077 Hong Kong

**Keywords:** alkali metal halide, domain distribution, perovskite light‐emitting diode, quasi‐2D perovskite

## Abstract

Solution processable quasi‐2D (Q‐2D) perovskite materials are emerging as a promising candidate for blue light source in full‐color display applications due to their good color saturation property, high brightness, and spectral tunability. Herein, an efficient energy cascade channel is developed by introducing sodium bromide (NaBr) in phenyl‐butylammonium (PBA)‐containing mixed‐halide Q‐2D perovskites for a blue perovskite light‐emitting diode (PeLED). The incorporation of alkali metal contributes to the nucleation and growth of Q‐2D perovskites into graded distribution of domains with different layer number <*n*>. The study of excitation dynamics by transient absorption (TA) spectroscopy confirms that NaBr induces more Q‐2D perovskite phases with small *n* number, providing a graded energy cascade pathway to facilitate more efficient energy transfer processes. In addition, the nonradiative recombination within the Q‐2D perovskites is significantly suppressed upon Na^+^ incorporation, as validated by the trap density estimation. Consequently, the optimized blue PeLEDs manifest a peak external quantum efficiency (*EQE*) of 7.0% emitting at 486 nm with a maximum luminance of 1699 cd m^−2^. It is anticipated that these findings will improve the understanding of alkali‐metal‐assisted optimization of Q‐2D perovskites and pave the way toward high‐performance blue PeLEDs.

## Graphical abstract

An efficient energy cascade channel is developed by introducing sodium bromide (NaBr) in phenyl‐butylammonium (PBA)‐containing halide perovskites. NaBr renders quasi‐2D perovskite films with graded domain distribution for efficient energy transfer, and contributes to enhanced radiative recombination. Consequently, the optimized PeLEDs manifest a peak external quantum efficiency of 7.0% at 486 nm with a maximum luminance of 1699 cd m^−2^.

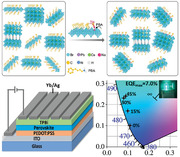

## 1 Introduction

Lead halide perovskites (LHP) have emerged as an exciting family of next‐generation optoelectronic materials owing to their fascinating features, including high defect‐tolerance, composition‐dependent bandgap tunability, high color‐purity, and facile fabrication through cost‐effective solution‐processing methods.^[^ ^1^, ^2^, ^3^, ^4^, ^5^ ^]^ The 3D structure of LHPs adopts a configuration of ABX_3_, where A refers to a small cation (such as, Cs^+^, FA^+^, or MA^+^), B refers to Pb^2+^, and X to the halide anion (Cl^−^, Br^−^, and I^−^). The 3D structure can be transformed into a quasi‐2D (Q‐2D) arrangement by introducing large spacer cations (S) and can be expressed as S_2_A*_n_*_−1_B*_n_* X_3_ *_n_*_+1_ (*n* represents the number of lead halide octahedral layers sandwiched between the spacer layers).^[^ ^4^, ^6^ ^]^ Dedicated efforts to harness the potential of Q‐2D perovskite for optoelectronics have led to the realization of high‐efficiency devices, such as solar cells, photodetectors, and light‐emitting diodes (LEDs) with state‐of‐the‐art performance.^[^ ^7^, ^8^, ^9^ ^]^ Particularly, perovskite LEDs (PeLEDs) with high luminance and external quantum efficiencies (EQEs)^[^ ^7^, ^10^, ^11^ ^]^ approaching to that of quantum‐dot and organic counterparts,^[^ ^12^, ^13^ ^]^ have been developed using Q‐2D perovskite structures. Moreover, owning to the good color tunability of Q‐2D perovskites, blue PeLEDs with the Commission Internationale de l'Eclairage (CIE) *y* and (*x*+*y*) coordinate values under 0.15 and 0.30, respectively, have been demonstrated, which are desired for high‐resolution displays and energy‐saving lighting.^[^ ^14^ ^]^ Nevertheless, the performance of blue PeLEDs remains inferior compared with their green and red/infrared counterparts, potentially due to the higher density of nonradiative recombination centers in large bandgap blue‐emitting LHPs.^[^ ^15^ ^]^ Currently, two common approaches are adopted to achieve blue emission, including i) bandgap engineering via mixing the halides (Br/Cl) in nanocrystals or 3D LHP films,^[^ ^16^, ^17^ ^]^ and ii) introducing long‐chain organic cations, for example, phenyl‐ethylammonium (PEA), butylammonium (BA), or phenyl‐butylammonium (PBA) as spacers to restrict the grain growth and produce 2D perovskites with strong quantum confinement.^[^ ^6^, ^15^ ^]^ To date, various approaches (e.g., A‐site cation engineering,^[^ ^11^ ^]^ interfacial modification,^[^ ^18^, ^19^ ^]^ or trap‐passivation^[^ ^16^ ^]^ methods) have been employed to develop blue PeLEDs. Notably, current high‐performing sky‐blue and blue PeLEDs are mostly realized by incorporating different types of long‐chain organic molecules in LHP emitters.^[^ ^15^ ^]^ Although this approach has enabled PeLEDs with decent performance, the insulating nature of these long‐chain organic spacers severely deteriorates the electronic properties of the resulting perovskite emitters and hence, the luminance of the corresponding devices.^[^ ^16^ ^]^ Moreover, Q‐2D perovskites are composed of multidimensional perovskite domains with various number of lead halide octahedral layers (e.g., *n* = 1, 2, 3, and 4).^[^ ^6^ ^]^ Among various Q‐2D domains, *n* = 1 is usually the most stable phase owing to its low formation energy and water resistance;^[^ ^20^ ^]^ however, this phase is believed to be dominated by severe nonradiative recombination because of the strong exciton−phonon coupling effect.^[^ ^21^, ^22^ ^]^ Therefore, it is imperative to control the growth of Q‐2D perovskites in such a way that *n* = 1 phase is suppressed and larger‐*n* phases (*n* = 2, 3, 4) with desired configuration are formed for effective utilization of excitons.

PeLEDs with improved performance have been manifested by incorporating various alkali‐metals beyond commonly used Cs (for instance, Rb and K) as the A‐site cations that contribute to a more stabilized perovskite structure with superior optoelectronic features.^[^ ^23^, ^24^, ^25^, ^26^ ^]^ Moreover, treating the substrates (usually, hole‐transport layers) with suitable alkali‐metal ions is found to be promising to reduce the interfacial defects for optimized growth of perovskite films that lead to the enhanced device performance.^[^ ^19^, ^20^, ^21^, ^22^, ^23^, ^24^, ^25^, ^26^, ^27^, ^28^ ^]^ Tang and co‐workers introduced potassium ions (K^+^) in poly(3,4‐ethylenedioxythiophene):poly(styrenesulfonate) (PEDOT:PSS) and the resulting K^+^‐modified substrate rendered perovskite films with high surface coverage and controlled crystal orientation, leading to an EQE of 4.14% with a maximum luminance of 451 cd m^−2^ for blue emission at 469 nm.^[^ ^19^ ^]^ To manipulate the domain distribution of Q‐2D perovskites to realize efficient energy funneling towards improved light‐emission, reports show that introduction of alkali‐metal halides can be a potential alternative to long‐chain organic spacers.^[^ ^29^, ^30^, ^31^ ^]^ For instance, Wu et al. have demonstrated the successful replacement of long‐chain organic molecules with NaBr that acted as an inorganic spacer ligand, to obtain Q‐2D perovskites for efficient green PeLEDs.^[^ ^29^ ^]^ This approach has also been extended to sky‐blue PeLEDs and it is observed that Na^+^ could suppress the formation of *n* = 1 phase while facilitating the growth of large‐*n* components (*n* = 2, 3 and 4) for efficient energy transfer.^[^ ^22^ ^]^ In a recent report, Zhou and colleagues investigated the role of different alkali‐metal salts, including LiBr, NaBr, and KBr on the formation of Q‐2D perovskites and attained efficient green PeLEDs with a peak EQE of 18.15% and luminance of 25 800 cd m^−2^ for their optimized device with K^+^‐incorporated perovskite emitters.^[^ ^32^ ^]^ The enhanced performance is attributed to the homogenous distribution of different *n*‐domains due to the coulombic interactions among alkali‐metal ions and the negatively charged PbBr_6_^4 −^octahedrons. The aforementioned studies indicate the suitability of alkali‐metals for the development of high‐performance PeLEDs; nonetheless, most of the previous reports are focused on the utilization of these candidates as A‐site cations, or additives for substrate modification while the impact of adopting alkali‐metals in Q‐2D LHPs is rarely explored. Therefore, a better understanding of the role of alkali‐metal‐assisted domain distribution of Q‐2D perovskites and underlying crystallization mechanism is desired to effectually tailor the energy transfer process for further improving the performance of blue PeLEDs.

Herein, we investigated the role of NaBr on the growth of PBA‐containing mixed‐halide Q‐2D perovskites for blue PeLEDs. The PBA^+^ as an organic spacer was introduced into the perovskite of pure CsPbBr_3_ to attain the Q‐2D perovskite films for sky‐blue emission. The NaBr is used as the spacer ligand to provide additional modulation of the distribution of the Q‐2D perovskite. We adopted a range of characterization tools to understand the NaBr‐assisted crystallization mechanism and the subsequent energy transfer processes. Grazing‐incidence small‐angle X‐ray scattering (GIWAXS) analysis illustrated the role of NaBr on the nucleation and growth of Q‐2D perovskites with graded distribution of different *n*‐domains. Time‐resolved photoluminescence (TRPL) spectroscopies revealed that the nonradiative recombination is significantly suppressed upon Na^+^ incorporation, as further validated by the trap density calculation. Consequently, the optimized blue PeLEDs by coordinating phase distribution manifested a peak EQE of 7.0% at 486 nm with a maximum luminance of 1699 cd m^−2^. These findings suggest that the strategy of managing phase distribution of Q‐2D CsPbBr_3_ thin films with combination of organic and sodium spacers could pave a promising way to obtain high efficiency blue PeLEDs.

## 2 Results and Discussion

**Figure 1:**
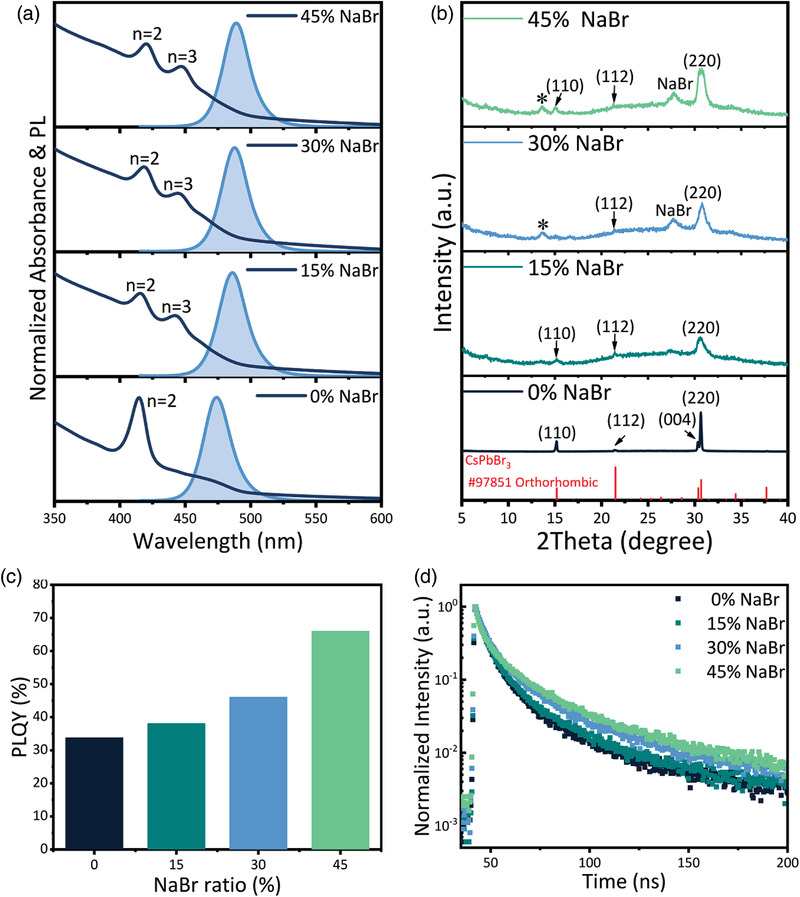
a) Normalized absorbance and PL spectra, and b) XRD patterns of Q‐2D CsPbBr_3_ perovskite films with various ratios of NaBr. c) PLQYs of corresponding Q‐2D perovskite films (excited at 1 mW cm^−2^), and d) PL lifetime traced at the emission peak of Q‐2D perovskite films with different NaBr contents under a fixed excitation power of 1 µJ cm^−2^ at an excitation wavelength of 365 nm.

The UV–vis absorption and PL spectra of perovskite films with different NaBr molar ratios were investigated. As shown in **Figure** **1**a, the Q‐2D perovskite film of 0.8 molar ratio PBACl without NaBr presents an obvious absorption peak at 415 nm, which is assigned to the *n* = 2 phase. The absorption spectra of the perovskite films with NaBr show a strong peak at 419 nm and a shoulder at 445 nm, which are associated with the *n *= 2 and *n *= 3 phase perovskites, respectively. When the molar ratio of NaBr was increased from 0% to 45%, the absorption edges were observed to monotonically red‐shift, and the corresponding PL peaks were tuned from 474 to 489 nm. Furthermore, the photoluminescence quantum yields (PLQYs) of Q‐2D perovskite films with various ratios of NaBr were also investigated to confirm their PL properties. As shown in Figure 1c, the NaBr molar ratio dramatically affects the PLQY of perovskite films. Pristine Q‐2D perovskite film showed a quite low PLQY value of 33.8% which slightly increased to 38.1% when 15% NaBr was added to the perovskite system. Upon further increasing the NaBr molar ratio, the PLQY has significantly improved to as high as ≈66% with 45% NaBr, resulting from the suppression of nonradiative recombination, which is likely caused by the surface passivation effect from NaBr.^[^ ^30^ ^]^

Moreover, to understand the influence of NaBr on the dynamics of photocarriers within Q‐2D perovskite films, time‐resolved PL were measured by time‐correlated single photon counting (Figure 1d). Tri‐exponential decay functions were used to fit the obtained PL decay curves, and the corresponding data are summarized in Table S1 (Supporting Information). The *τ*_1_ could be ascribed to the fast process related to Auger recombination or trap‐assisted charge recombination, *τ*_2_ corresponds to the bimolecular radiative recombination process, and *τ*_3_ is attributed to the slow radiative recombination process.^[^ ^22^, ^33^ ^]^ Accordingly, *A*_1,_ *A*_2_, and *A*_3_ are the fractions of the fast (*τ*_1_), middle (*τ*_2_), and slow (*τ*_3_) decay components, respectively. The extracted average PL lifetime (*τ*_ave_) of ≈35 ns for pristine perovskite film progressively raised to 57 ns as the NaBr ratio is increased from 0% to 45%. The longer PL lifetime generally indicates a lower defect density and more efficient radiative recombination, which accounts for the PLQY enhancement as shown in Figure 1c.^[^ ^34^ ^]^

Furthermore, we calculated the radiative and nonradiative recombination rate constants, referred as *k*_rad_ and *k*_nonrad_, respectively, of the fabricated perovskite films. The decay time implies the average lifetime (*τ*_avg_) that includes both *k*_rad_ and *k*_nonrad_ and can be determined by^[^ ^35^, ^36^ ^]^:

(1) $$\frac{1}{\tau_{\mathrm{avg}}}=k_{\mathrm{rad}}+k_{\mathrm{nonr}\mathrm{ad}}$$

The PLQY is described as the rate of radiative recombination divided by the total rate of recombination:

(2) $$\mathrm{PLQY}=\frac{k_{\mathrm{rad}}}{k_{\mathrm{rad}}+k_{\mathrm{nonr}\mathrm{ad}}}$$

Hence, the above two mathematical relationships lead to:

(3) $$k_{\mathrm{rad}}=\frac{\mathrm{PLQY}}{\tau_{\mathrm{avg}}}$$

The *k*_rad_ and *k*_nonrad_, and related optoelectronic parameters of the perovskite films are summarized in **Table** **1**. Compared to the pristine perovskite film, NaBr incorporated counterparts exhibited longer *τ*_avg_ as a consequence of improved radiative recombination; this enhanced *k*_rad_ and substantially suppressed *k*_nonrad_ in the NaBr‐containing perovskite films may have resulted in their higher PLQYs, owing to the more effectively confined excitons for light‐emission with the incorporation of NaBr.^[^ ^37^, ^38^ ^]^ Taken together, both the PLQY values and PL lifetimes show that the incorporation of NaBr can substantially suppress the nonradiative recombination in perovskite films, making them suitable as emissive layers for LED.

**Table 1:** Optoelectronic parameters of perovskite films with various ratios of NaBr

Perovskite Emitter	PL Peak [nm]	PLQY [%]	*τ*_ave_ [ns]	*k*_rad_ [s^−1^]	*k*_nonrad_ [s^−1^]	*k*_rad_ *:k*_nonrad_
0% NaBr	478	33.8	34.82	9.7 × 10^6^	1.9 × 10^7^	0.51
15% NaBr	485	38.1	40.27	9.5 × 10^6^	1.5 × 10^7^	0.62
30% NaBr	485	46.1	49.11	9.4 × 10^6^	1.1 × 10^7^	0.86
45% NaBr	487	66	57.20	1.2 × 10^7^	5.9 *** *** × 10^6^	1.94

In metal halide perovskites, trap‐assisted recombination, which relates to the trap density in perovskite films, is a typical nonradiative recombination process.^[^ ^39^, ^40^ ^]^ According to Figure S1 (Supporting Information), the trap density in the pristine Q‐2D perovskite film is about 1.03 × 10^18^ cm^−3^, causing a low PLQY of around 34%. Encouragingly, the trap density of resulting perovskite films dramatically reduced from ≈1.03 × 10^18^ to 6.73 × 10^17^ cm^−3^ as the ratio of NaBr was increased from 0 to 45%. Therefore, the introduction of the NaBr in as‐obtained Q‐2D films has substantially reduced the trap density and suppressed the trap‐assisted nonradiative recombination, which is critical for efficient blue emission.

To further study the crystallization mechanism of the perovskite films after NaBr addition, X‐ray diffraction (XRD) measurement was performed and the obtained patterns are shown in Figure 1b. In the case of pristine Q‐2D film, the diffraction peaks are observed at 15.2°, 21.5°,30.3°, and 30.7°, which respectively correspond to the lattice planes of (110), (112), (004), and (220) of the orthorhombic CsPbBr_3_ phase.^[^ ^48^ ^]^ After the incorporation of varied NaBr ratios, the main diffraction peaks of the orthorhombic CsPbBr_3_ phase became broader and weaker, indicating a gradual disruption of the ordered crystal stacking of CsPbBr_3_ with the introduction of the NaBr spacer.^[^ ^22^, ^29^, ^49^ ^]^ Moreover, for 30% and 45% NaBr cases, the XRD peak marked by the star at 27.7° matches‐well with the NaBr crystallization, indicating the aggregation of excess NaBr.

**Figure 2:**
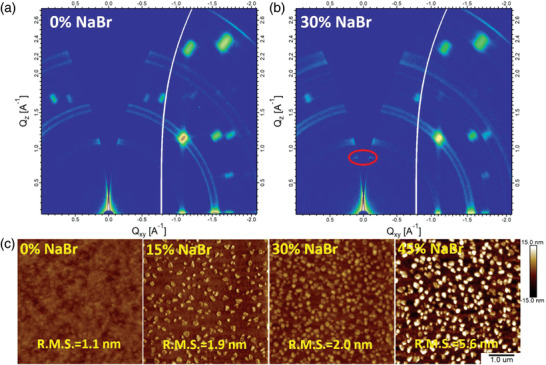
GIWAXS patterns of a) pristine and b) 30% NaBr‐incorporated Q‐2D perovskite film. c) Atomic force microscope (AFM) images of Q‐2D perovskite films with various ratios of NaBr. All AFM images are 5 µm^2^.

It is widely established that the resulting film morphology are other critical factors that govern the final performance of PeLEDs. We therefore employed scanning electron microscopy (SEM) and atomic force microscopy (AFM) to unveil the morphological evolution of Q‐2D films upon introducing NaBr in different ratios. As shown in **Figure** **2**c and Figure S2 (Supporting Information), pristine Q‐2D perovskite film exhibited full coverage and low root‐mean square (RMS) roughness of 1.1 nm with an average grain size of above 100 nm. After the introduction of NaBr in a concentration range of 15–45% into perovskite the precursor solution, films with high surface‐coverage and smaller nanocrystals were successfully formed, resulting in a low RMS roughness of ≈2 nm. However, with NaBr ratio exceeding 30%, a few tiny particles are observed on the surface of the film and the RMS roughness of perovskite film was increased to 5.6 nm, which may be ascribed to the aggregation of excess NaBr in perovskite film. X‐ray photoelectron spectroscopy (XPS) measurements were performed to further trace the location of Na^+^ in Q‐2D perovskites. As shown in Figure S4a (Supporting Information), the XPS spectra confirm the presence of Cs, Pb, Cl, Br, and N elements in both, pristine and optimized perovskite films. Moreover, the spectra of Na 1s in Figure S4b (Supporting Information) certify the presence of Na^+^ in the NaBr‐incorporated film. The two obvious peaks of Pb 4f ≈138.5 and 143.4 eV in the XPS spectra (Figure S4c, Supporting Information) correspond to the Pb 4f7/2 and Pb 4f5/2 levels of Pb−Cl and Pb−Br, respectively. Also, the XPS spectra of Cs 3d are shown in Figure S4c (Supporting Information). Both the XPS spectra of Pb 4f and Cs 3d show no shift with or without NaBr, which further demonstrates that Na^+^ has no effect on the internal crystal structure of perovskite, which is consistent with XRD results as shown in Figure 1b.

**Figure 3:**
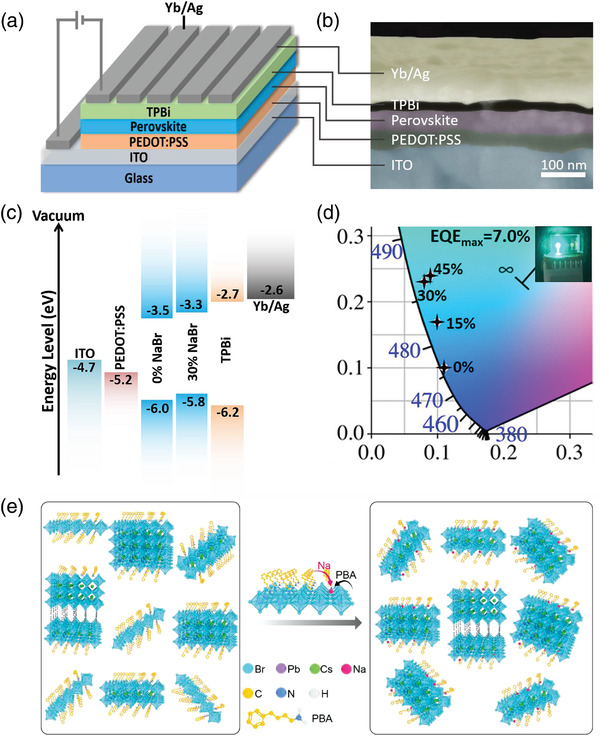
a) Schematic device structure and b) cross‐sectional SEM image of the as‐fabricated PeLED. c) Corresponding energy‐levels diagram. d) Commission Internationale de lQEclairage (CIE) coordinates of the fabricated PeLEDs. The photo in the inset displays a working blue PeLED with 30% NaBr at 100 cd m^−2^. (e) Schematic illustrating the impacts of Na^+^ on the phase distribution of Q‐2D perovskites.

GIWAXS was further utilized to investigate the crystal structure and orientation of the fabricated perovskite films. As shown in Figure 2a,b, the diffraction rings are present in the GIWAXS patterns of both pristine and 30%‐NaBr incorporated films, indicating that the 3D grains in polycrystalline perovskite films were oriented in random directions.^[^ ^47^ ^]^ An extra diffraction dot (marked with a red circle) at (*q*_xy_ = 0 Å^−1^, *q*_z_ = 0.88 Å^−1^) was observed in the small *q*‐value region for 30%‐NaBr incorporated film. The calculated *d*‐spacing of 7.1 Å is consistent with the lattice parameter of the 2D perovskite diffraction peak (2*θ* = 13.6°) in XRD patterns. To our knowledge, 3D CsPbBr_3_ crystallizes in a cubic space group with dimensions of *a* = *b* = *c* = 5.83 Å, and one [PbBr_4_]^2−^ octahedral lattice size is 5.83 Å. The radius of sodium is 1.16 Å if it is in 6‐coordinate state. The distance of 7.1 Å ((5.83 × 2+1.16 × 2)/2 = 6.99) is ascribed to the (020) plane between discrete Ruddlesden–Popper (RP) Na_2_CsPb_2_Br_7_ nanoplatelet.^[^ ^29^ ^]^ Besides, it is worth noting that the films containing NaBr are not pure 2D perovskite, we can also observe diffraction dots that are corresponding to the 3D bulk perovskite phase but with better crystal orientation compared with the pristine film. So a better oriented 2D/3D mixed phase instead of pure 2D phase is formed in the case of NaBr incorporated film. We attribute the observed results to various factors, including i) quite low content of the organic spacer and hence, consequent formation of 2D/Q‐2D phases in low density,^[^ ^50^ ^]^ and/or ii) 2D perovskite phase generally does not possess a periodic crystal arrangement, causing low diffraction intensity.^[^ ^51^ ^]^ Therefore, it is very likely that a 2D/3D hybrid perovskite is formed in the NaBr incorporated films. We show a schematic diagram illustrating the impacts of Na^+^ on the phase distribution of Q‐2D perovskites in **Figure** **3**e.

**Figure 4:**
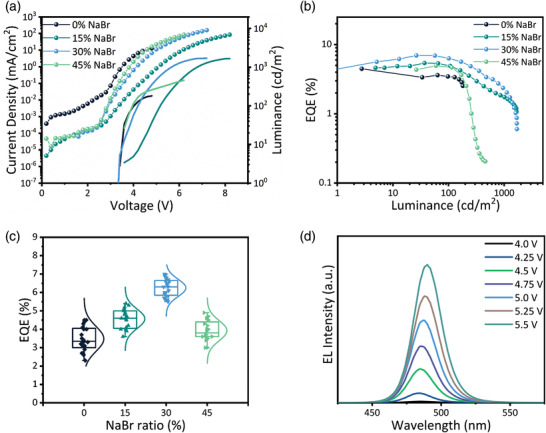
Performance of PeLEDs adopting perovskite films with different NaBr ratios a) Current density and luminance versus voltage. Dotted lines and solid lines correspond to current density and luminance, respectively. b) EQE versus luminance curves. c) Statistics of maximum EQEs of 20 devices for each condition. d) EL spectra of PeLED with 30% NaBr under various applied voltages.

To evaluate the electroluminescence (EL) performance of NaBr‐incorporated Q‐2D perovskite films, PeLEDs with a device structure of indium tin oxide (ITO)/PEDOT:PSS/Q‐2D CsPbBr_3_ Perovskite/TPBi/Yb/Ag were fabricated (Figure 3a), where PEDOT:PSS (CH8000) with a deep work function of −5.15 eV works as the hole injection layer, TPBi is introduced as an electron transport layer, and Yb acts as a cathode interface layer for efficient electron injection from Ag.^[^ ^41^ ^]^ The valance band (VB) of perovskite films (pristine and 30% NaBr‐incorporated samples) were estimated by combining the ultraviolet photoelectron spectroscopy (UPS) results (Figure S5, Supporting Information) with the optical bandgaps obtained from the absorption spectra (Figure 1a). As shown in the energy‐levels diagram (Figure 3c) and UPS results (Figure S5, Supporting Information), the Fermi levels are close to VBs of perovskite films, which suggest *p*‐type characteristic of the films.^[^ ^42^, ^43^ ^]^ The addition of NaBr leads to a shift‐up of the energy levels, presenting a better energy‐level alignment for both electron and hole injection to the emissive layer. Figure S6 (Supporting Information) shows the EL spectra of PeLEDs with different ratios of NaBr. All the studied PeLEDs showed narrow full‐widths at half‐maximum (FWHMs) of <27 nm for EL emission, confirming high color purity in CIE coordinates, as shown in Figure 3d. The performance of Q‐2D PeLEDs with various concentrations of NaBr is summarized in **Table** **2** and displayed in Figures 4a,b.

**Table 2:** Device performance summary for PeLEDs with various ratios of NaBr

NaBr Ratio	V_on_ [V]	L_max_ [cd m^−2^]	CE_max_ [cd A^−1^]	*EQE*_max_ [%]	CIE [x,y]	EL Peak [nm]	FWHM [nm]
0%	3.3	180	3.2	4.5	(0.11,0.10)	475	22
15%	3.4	1672	6.5	5.4	(0.10,0.17)	482	26
30%	3.3	1699	9.0	7.0	(0.08,0.23)	486	24
45%	3.5	459	6.6	4.9	(0.09,0.24)	487	27

PeLED adopting pristine Q‐2D perovskite film suffered from a large leakage current and manifested a low EQE of 4.5%. Upon raising the NaBr ratio in Q‐2D perovskite films from 0% to 30%, the leakage current was considerably suppressed and the device performance was dramatically improved; our champion PeLED attained a high EQE of 7.0% with relatively low efficiency roll‐off. However, further increasing the NaBr ratio to 45% reduced the EQE and luminance while the device showed significant efficiency roll‐off; this inferior performance comparing to that of the 30% NaBr‐based PeLED could be due to the reduced crystal quality of corresponding film. These results reveal that the trend of EQE change with increasing NaBr ratios is almost similar to that of the PLQYs of corresponding perovskite films, suggesting that the EL performance of PeLEDs is governed by the PL characteristics of perovskite films. While, the EQE of 45% NaBr based PeLEDs had dropped, resulting from the excess NaBr aggregation. Also, the PeLED performance is strongly affected by the trap densities in perovskite films as higher trap density could result in severe trapping of the injected charge carriers and hence, causing severe trap‐assisted nonradiative recombination. Statistics of maximum EQEs of 20 devices for each case are displayed in **Figure** **4**c. PeLEDs with 30% NaBr showed a relative standard deviation of 37% for the peak efficiency, which is noticeably reduced comparing to that of 68% for the pristine counterparts, suggesting that improved reproducibility can be realized through optimizing the film features.

In addition, a gradual red‐shift of the EL from 475 to 487 nm was observed with an increment in the NaBr ratio (Figure S6, Supporting Information), which correlates‐well with the red‐shift of PL spectra. With the red‐shift of EL spectra, the corresponding CIE coordinates also changed from blue to the sky‐blue region. In addition, a slight red‐shift in EL spectra was observed at different applied voltages for the optimized device (with 30% NaBr) as can be seen in Figure 4d, indicating excellent spectral stability of the device. The operational lifetimes, which are measured at the constant current density with an initial luminance of 100 cd m^‐2^, are shown in Figure S9 (Supporting Information). The optimized device with 30% NaBr shows longer half‐lifetime comparing to the control device without NaBr, demonstrating that the operational stability is improved after defect passivation.

**Figure 5:**
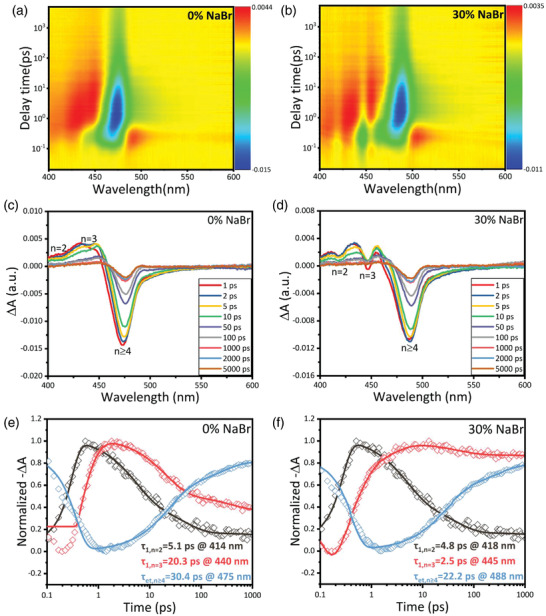
Pseudocolour transient absorption (TA) spectrum plot of a) pristine and b) 30% NaBr‐incorporated perovskite films. Corrected femtosecond TA spectra of c) pristine and d) 30% NaBr‐based perovskite films at selected probe delay times. TA spectra at different wavelength as a function of delay time for e) pristine and f) 30% NaBr‐based perovskite films respectively. Solid lines are the fits of the kinetics by exponential function with fitting parameters listed in Tables S2 and S3 (Supporting Information) respectively.

Transient absorption (TA) spectroscopy was employed to investigate the carrier transport and recombination process of photogenerated charges in Q‐2D perovskite films with various ratios of NaBr. For TA measurements, a pump pulse was used to excite the perovskite films and the induced absorption changes (Δ*A*) were recorded to track the recovery of the transient bleach of the band edge absorption. **Figure** **5**a,b displays the pseudo‐color TA plot and Figure 5c,d demonstrates the TA spectra at different selected delay times for the pristine and 30% NaBr‐incorporated perovskite films. Intriguingly, three distinctive bleach peaks are observed at 414 nm (*n* = 2), 440 nm (*n* = 3), and 475 nm (*n* ≥ 4) for the pristine Q‐2D perovskite films (Figure 5a–c), while at 418 nm (*n* = 2), 445 nm (*n *= 3) and 488 nm (*n* ≥ 4) for the 30% NaBr‐based perovskite films (Figure 5b–d). The peak positions of bleaches are in good agreement with peaks in the steady‐state absorption spectra (Figure 1a) and EL peaks in Table 2, indicating the EL peaks at 475 nm for 0% NaBr based PeLEDs and 486 nm for 30% NaBr based PeLEDs inherently are emitted through *n* ≥ 4 phase. With an increase of decay time, the intensity of ground‐state bleaching (GSB) for *n* = 2 and *n* = 3, i.e., GSB*_n_*_= 2_ and GSB*_n_*_= 3_ gradually disappeared while that of the GSB*_n_*_≥ 4_ weakens, indicating that an energy transfer process takes place and the charge‐carriers transfer from donor domains (*n *= 2 and 3) to the emitting domain (*n* ≥ 4) is accomplished in picoseconds. These results suggest that a cascade energy transfer from high‐energy domains to lower‐energy ones happened in these perovskite films on a timescale of 100 fs to 100 ps. After ≈100 ps, the downward funnelling of energy is almost completed and the remaining dynamics correspond to recombination in the lowest‐energy domains. Each bleach recovery dynamic of the pristine and 30% NaBr‐incorporated perovskite films is extracted and well‐fitted by multiexponential function in Figure 5e and f (fitting parameters are listed in Tables S2 and S3, Supporting information). The fast decay components (*τ*_1_) for *n* = 2 and 3 bleaching are corresponded to the carrier transfer from lower‐*n* species to the emitting *n* ≥ 4 domain species. As shown in Figure 5e,f, the fast decay component time constants (*τ*_1_) of 4.8 ps (*n* = 2) and 2.5 ps (*n* = 3) in 30% NaBr‐incorporated film is shorter than 5.1 ps (*n* = 2) and 20.3 ps (*n* = 3) in the case of pristine film. Coincidentally, the fitted time constants (*τ*_et_) of GSB*_n_*_≥ 4_ are 22.2 and 30.4 ps for the 30% NaBr‐incorporated and pristine perovskite films respectively, which accounts for the energy accepting from low‐*n* domains to *n* ≥ 4 domains. Faster energy transfer means less energy loss during the transfer process and further higher carrier concentration in the emitting centres. Obviously, the optimized film with 30% NaBr exhibited less carrier trapping and more radiative recombination, further proving that the addition of sodium greatly suppressed the carrier trapping and eventually provided a more efficient radiative recombination.^[^ ^34^, ^45^ ^]^ In summary, these studies of excitation dynamics suggest that the incorporation of NaBr induced more efficient energy transfer and improved homogeneous energy landscape towards the smallest bandgap emitters.

In conclusion, we have thoroughly investigated the role of NaBr on the growth of PBA‐containing mixed‐halide Q‐2D perovskites for blue PeLEDs. We adopted a range of characterization tools to study the NaBr‐assisted crystallization mechanism and subsequent energy transfer process. GIWAXS analysis clearly illustrated the beneficial role of NaBr on the nucleation and growth of Q‐2D perovskites into graded distribution of different *n*‐domains. Temperature‐dependent PL and TRPL spectroscopies revealed that the nonradiative recombination is significantly suppressed upon Na^+^ incorporation, as further validated by the trap density estimation. The study of excitation dynamics by TA spectroscopy confirmed that NaBr induced more small‐*n* Q‐2D perovskite phases, providing a graded domain distribution for cascade energy transfer toward large‐*n* species. Consequently, our optimized blue PeLEDs manifested a peak EQE of 7.0% at 486 nm with a maximum luminance of 1699 cd m^−2^. We anticipate that our findings will improve the understanding about alkali‐metal‐assisted Q‐2D perovskite optimization and would promote the development of efficient blue PeLEDs.

## 3 Experimental Section

### Materials

CsBr (99.999%), NaBr (99.99%) and PbBr_2_ (99.999%) were purchased from Sigma–Aldrich. PBACl (99.5%) was obtained from Xi'an Polymer Light Technology Corp. DMSO (anhydrous, >99.0%) was acquired from TCI. TPBi and Yb were procured from Lumtec and Alfa Aesar, respectively. PEDOT:PSS (CH 8000) was attained from Heraeus. All chemicals were used as received without further purification.

### Perovskite Precursor Preparation

PbBr_2_ (0.25 mmol) and CsBr were dissolved in 1 mL DMSO to form a 0.25 m CsPbBr_3_ perovskite precursor solution. Appropriate amounts of PBACl and NaBr were dissolved in the resulting CsPbBr_3_ solutions to form 0.25 m perovskite precursor solution with different ratios of NaBr. The molar ratio of PBACl to CsPbBr_3_ solutions remained as 0.8 in all the fabricated perovskite films while the concentration of NaBr was varied. The ratio of x% NaBr refers to the molar ratio between NaBr and PbBr_2_ (i.e., *m*_NaBr_/*m*_PbBr2_ = x%). The precursor solution was stirred overnight without any heating.

### Film Characterization

UV–vis absorption spectra were measured with HP8453 spectrophotometer. The PL spectra were collected with a spectrofluorometer (Perkin‐Elmer LS 55). AFM (Digital Instrumental [DI] Multimode Nanoscope IIIa) and SEM (ZEISS Merlin) measurements were carried out based on a structure of ITO/PEDOT/perovskite. XRD measurement was carried out based on the structure of glass/PEDOT:PSS/perovskite by an X‐ray diffractometer (PANalytical X'pertPRO) equipped with Cu‐K*α* X‐ray tube. The XPS spectra were collected on Thermo Fisher Scientific Escalab 250 Xi equipment. PLQY values were obtained from perovskite films on glass at an excitation wavelength of 365 nm using a calibrated integrating sphere. Time‐resolved PL lifetime measurements were performed with a transient photoluminescence spectrometer (FLS980, Edinburgh Instruments) equipped with a time‐correlated single‐photon counting unit. A picosecond laser diode (365 nm, pulse width = 50 ps) was used as the excitation source. The fs‐TA measurements were performed on a Helios pump‐probe system (Ultrafast Systems LLC) coupled with an amplified femtosecond laser system (Coherent, 35 fs, 1 kHz, 800 nm). The probe pulses (from 380 to 600 nm) were generated by focusing a small portion (≈10 µJ) of the fundamental 800 nm laser pulses into a 1 mm CaF_2_. The 365 nm pump pulses were generated from an optical parametric amplifier (TOPAS‐800‐fs). GIWAXS patterns were measured on beamline 1W2A at South China University of Technology (SCUT), China. A monochromatic beam of *λ* = 1.54 Å was used, and the incident angle was 0.2°. The UPS spectra were collected on Thermo Scientific ESCALab 250Xi using a He I ultraviolet radiation source (21.22 eV).

### Perovskite LED Device Fabrication

The patterned ITO‐coated glass substrates were cleaned subsequently by sonication in detergent, acetone, deionised water, and isopropyl alcohol and then dried at 65 °C in a baking oven. After 4 min of oxygen plasma treatment, diluted PEDOT:PSS (CH 8000) was spin‐coated on ITO‐coated glass substrate at 4000 rpm for 40 s and then annealed at 150 °C for 15 min in ambient air. Perovskite precursor solution was then spin‐coated on PEDOT:PSS film at 3000 rpm for 60 s. Subsequently, the obtained perovskite films were annealed at an optimized temperature of 80 °C for 5 min in a N_2_‐filled glovebox. A 30 nm thick TPBi was evaporated onto the perovskite layer, followed by the deposition of Yb (5 nm) and Ag (100 nm) by thermal deposition in a vacuum chamber (pressure **≈**2 **× **10**^−^** ^6^ Torr). The device's active area was 0.045 cm^2^.

### Perovskite Device Characterization

Current density‐voltage‐radiance measurement was carried out with a Keithley 2400 source measurement unit and a Konica Minolta Chroma Meter CS‐200. The electroluminescence spectra and CIE coordinates were recorded with an Ocean Optics USB 2000+ spectrometer. The external quantum efficiency values were calculated assuming a Lambertian emission profile. The trap densities of the perovskite films were extracted by the dark current–voltage characteristics of the hole‐only devices in the device architecture of ITO/PEDOT:PSS/perovskite/MoO_3_ (10 nm)/Ag (100 nm) through a computer‐controlled Keithley 2400 source meter.

## Conflict of Interest

The authors declare no conflict of interest.

## Supporting information

Supporting Information

## Data Availability

The data that support the findings of this study are available from the corresponding author upon reasonable request.
