# Supplementary material for: Unravelling Alkali‐Metal‐Assisted Domain Distribution of Quasi‐2D Perovskites for Cascade Energy Transfer toward Efficient Blue Light‐Emitting Diodes
Source: Adv Sci (Weinh). 2022 May 13;9(20):2200393. doi: 10.1002/advs.202200393 (PMC9284168; doi:10.1002/advs.202200393)
Supplement: Supplementary file 1 — Supporting Information [file ADVS-9-2200393-s001.pdf]

ADVANCED SCIENCE
Open Access

Supporting Information

for *Adv. Sci.*, DOI 10.1002/advs.202200393

Unravelling Alkali-Metal-Assisted Domain Distribution of Quasi-2D Perovskites for Cascade Energy Transfer toward Efficient Blue Light-Emitting Diodes

Wanqing Cai, Muhammad Umair Ali, Ping Liu, Miao He, Cong Zhao, Ziming Chen, Yue Zang, Man-Chung Tang, Hong Meng, Hongyan Fu, Guodan Wei* and Hin-Lap Yip**

Unravelling Alkali-Metal-Assisted Domain Distribution of Quasi-2D Perovskites for Cascade Energy Transfer towards Efficient Blue Light-Emitting Diodes

Wanqing Cai[1,2], Muhammad Umair Ali[1,2], Ping Liu[1,2], Miao He[1,2], Cong Zhao[1,2], Ziming Chen[3], Yue Zang[3], Man-Chung Tang[1,2], Hong Meng[4], Hongyan Fu[1,2,*], Guodan Wei[1,2*] and Hin-Lap Yip[3,5,6*]

[1] *Tsinghua-Berkeley Shenzhen Institute (TBSI), Tsinghua University, Shenzhen 518055, China*

[2] *Tsinghua Shenzhen International Graduate School, Tsinghua University, Shenzhen City, 518055, China*

[3] *State Key Laboratory of Luminescent Materials and Devices, Institute of Polymer Optoelectronic Materials and Devices, School of Materials Science and Engineering, South China University of Technology, 381 Wushan Road, Guangzhou 510640, P. R. China*

[4] *School of Advanced Materials, Peking University Shenzhen Graduate School, Shenzhen City, 518055, P. R. China*

[5] *Department of Materials Science and Engineering, City University of Hong Kong, Kowloon, 999077 Hong Kong*

[6] *School of Energy and Environment, City University of Hong Kong, Kowloon, 999077 Hong Kong*

*Corresponding Authors:

Email: hyfu@sz.tsinghua.edu.cn; weiguodan@sz.tsinghua.edu.cn; a.yip@cityu.edu.hk

Supporting Information

Table S1. Summary of the tri-exponential fitting results for PL lifetime curves of Q-2D $CsPbBr_3$ films with various NaBr concentrations. Time-resolved PL lifetime curves were fitted by the tri-exponential function:

$$I = A_1 \exp\left(-\frac{t}{\tau_1}\right) + A_2 \exp(-\frac{t}{\tau_2}) + A_3 \exp(-\frac{t}{\tau_3}) \quad \textbf{(S1)}$$

Where, I is the normalized photoluminescence intensity; A_1, A_2 and A_3 are the fractions of the three decay components and $A_1 + A_2 + A_3 = 1$; τ_1, τ_2 and τ_3 stand for the lifetime constants of a fast

component, a middle component and a slow component, respectively. The average lifetime (τ_{ave}) was calculated with the A_i and τ_i (i= 1, 2, 3) values according to the following equation:

$$\tau_{ave} = \frac{A_1\tau_1 + A_2\tau_2 + A_3\tau_3}{A_1 + A_2 + A_3} \quad \textbf{(S2)}$$

Perovskite Emitter	Emission [nm]	τ_1 [ns]	A_1 [%]	τ_2 [ns]	A_2 [%]	τ_3 [ns]	A_3 [%]	τ_{ave} [ns]	χ^2
0% NaBr	478	2.20	13.21	10.87	38.82	40.51	47.97	34.82	1.07
15% NaBr	485	3.27	30.21	12.01	46.02	55.01	23.77	40.27	1.10
30% NaBr	485	3.64	34.12	12.88	45.40	68.30	20.48	49.11	1.12
45% NaBr	487	4.16	26.82	16.49	46.94	75.97	26.24	57.20	1.15

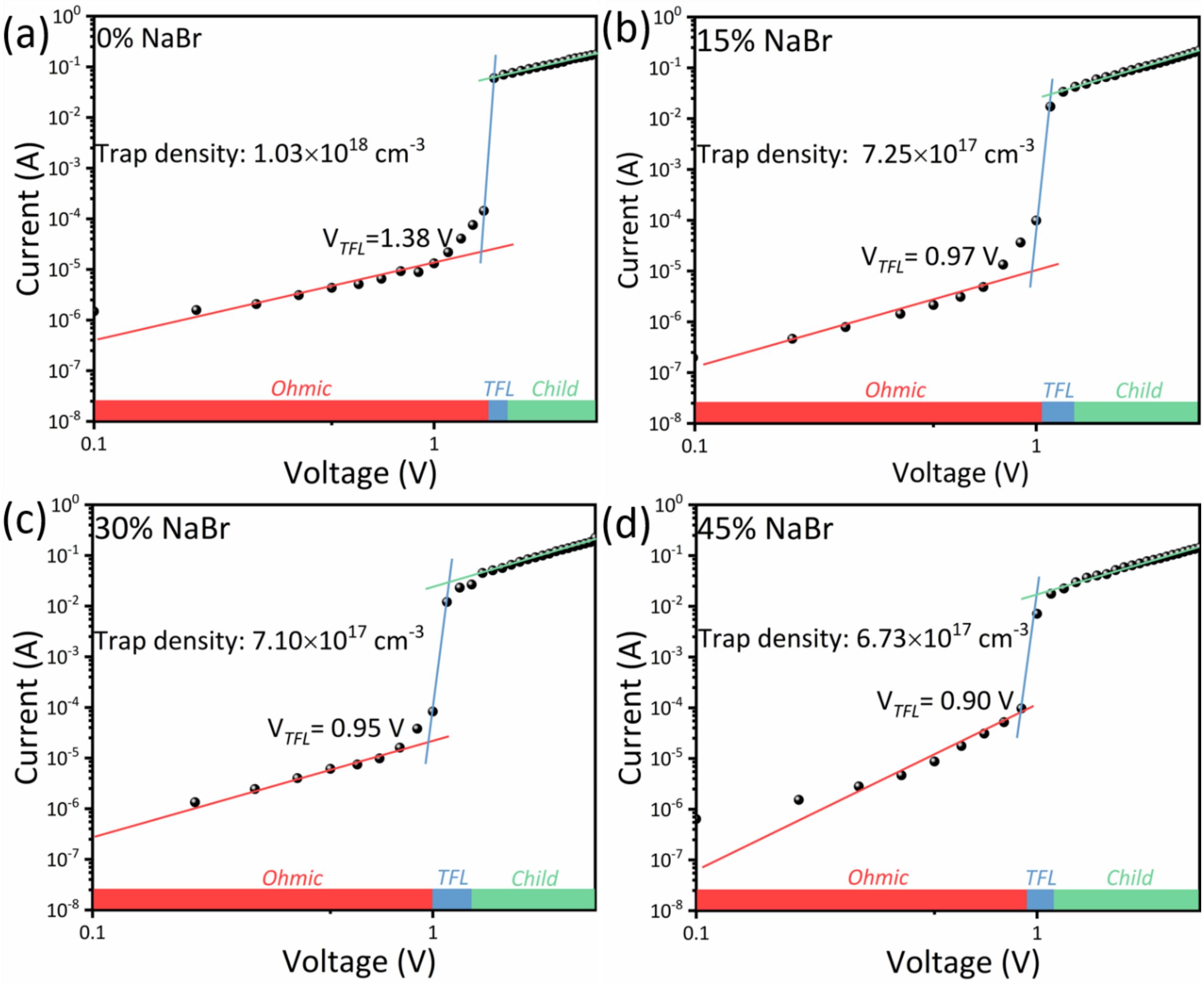

Figure S1. Trap density extraction by dark current-voltage measurement of the hole-only device, with the device structure of ITO/PEDOT/perovskite/MoO_3/Ag. The light-blue lines represent the ohmic regime of each case, and the dark-blue lines indicate the trap-filled limit (TFL) regime with the onset voltage (V_{TFL}). The trap density (N_t) of Q-2D perovskite films with various ratios of NaBr can be extracted by the equation $N_t=\frac{2\varepsilon_0\varepsilon_r VTFL}{qL2}$, where ε_0, q and L represent vacuum permittivity, elementary charge and thickness of the perovskite film, respectively; ε_r is the average relative dielectric constant of Q-2D perovskite films with various ratios of NaBr.

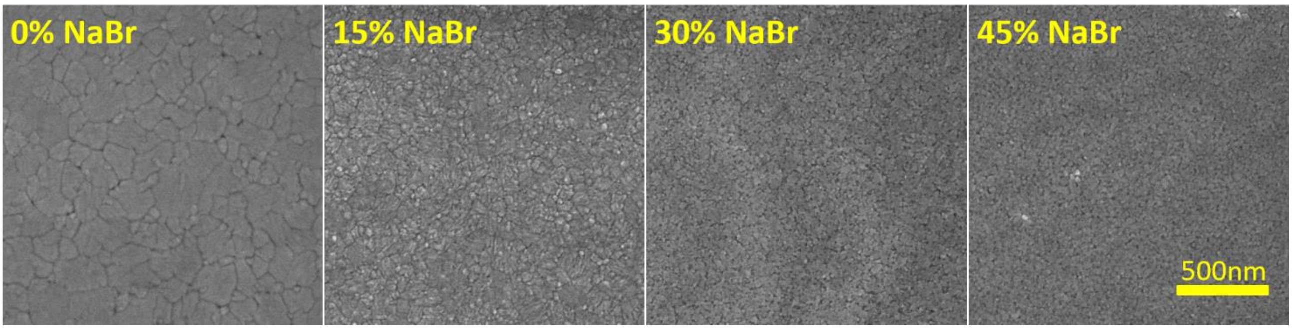

Figure S2. Scanning electron microscope (SEM) images of Q-2D perovskite films with various ratios of NaBr.

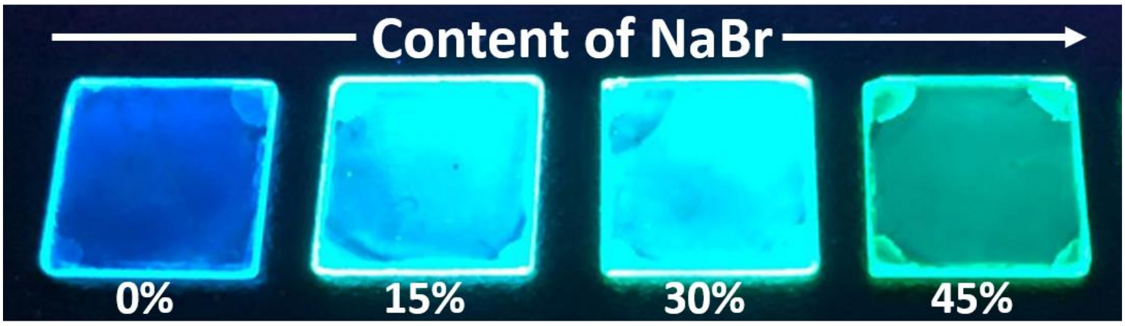

Figure S3. Photoluminescence image of quasi-2D perovskite films with various ratios of NaBr under ultraviolet lamp excitation (365 nm).

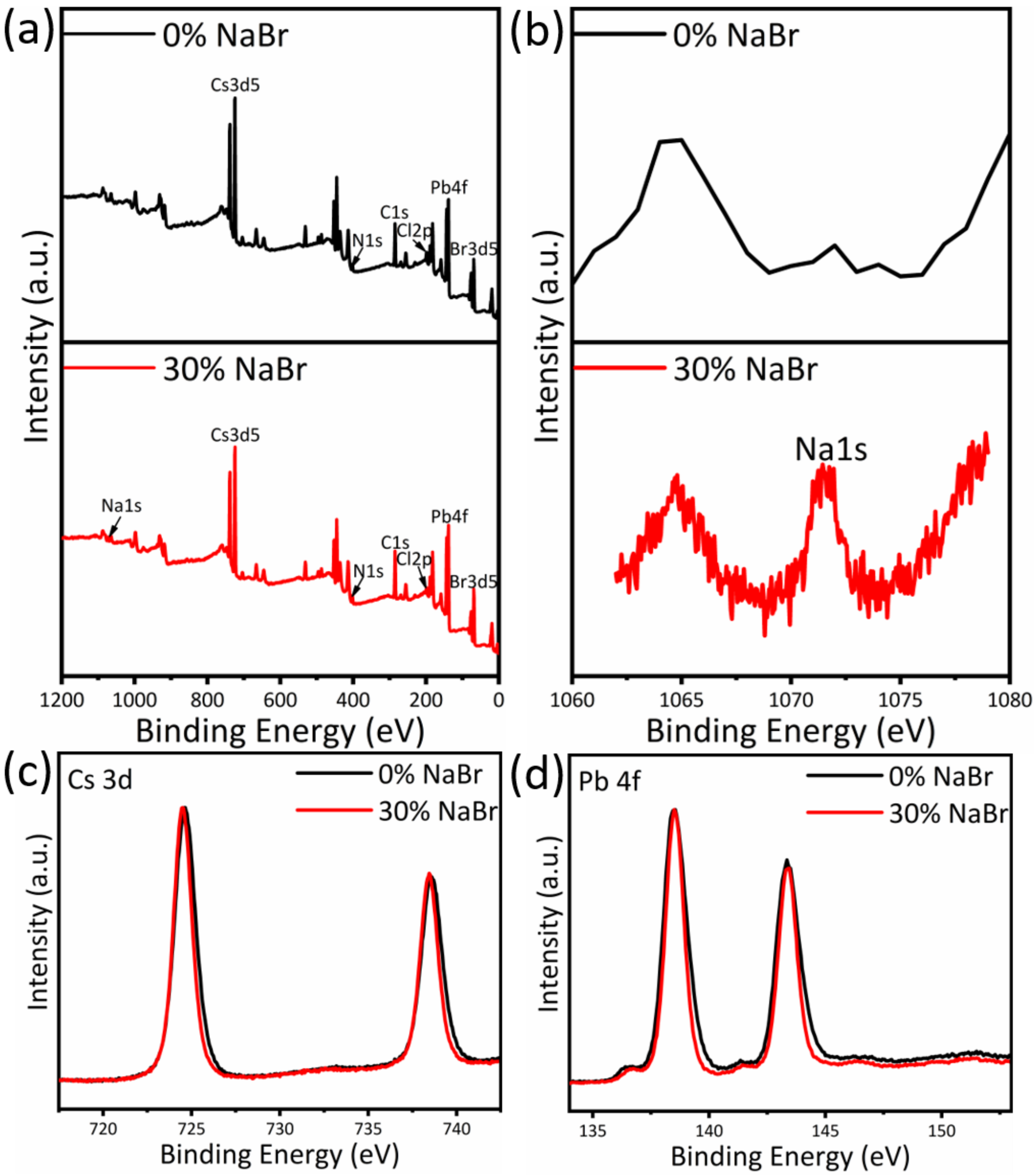

Figure S4. XPS spectra of perovskite films without and with 30% NaBr: (a) Entire spectra, (b) Na 1s spectra, (c) Cs 3d spectra and (d) Pb 4f spectra.

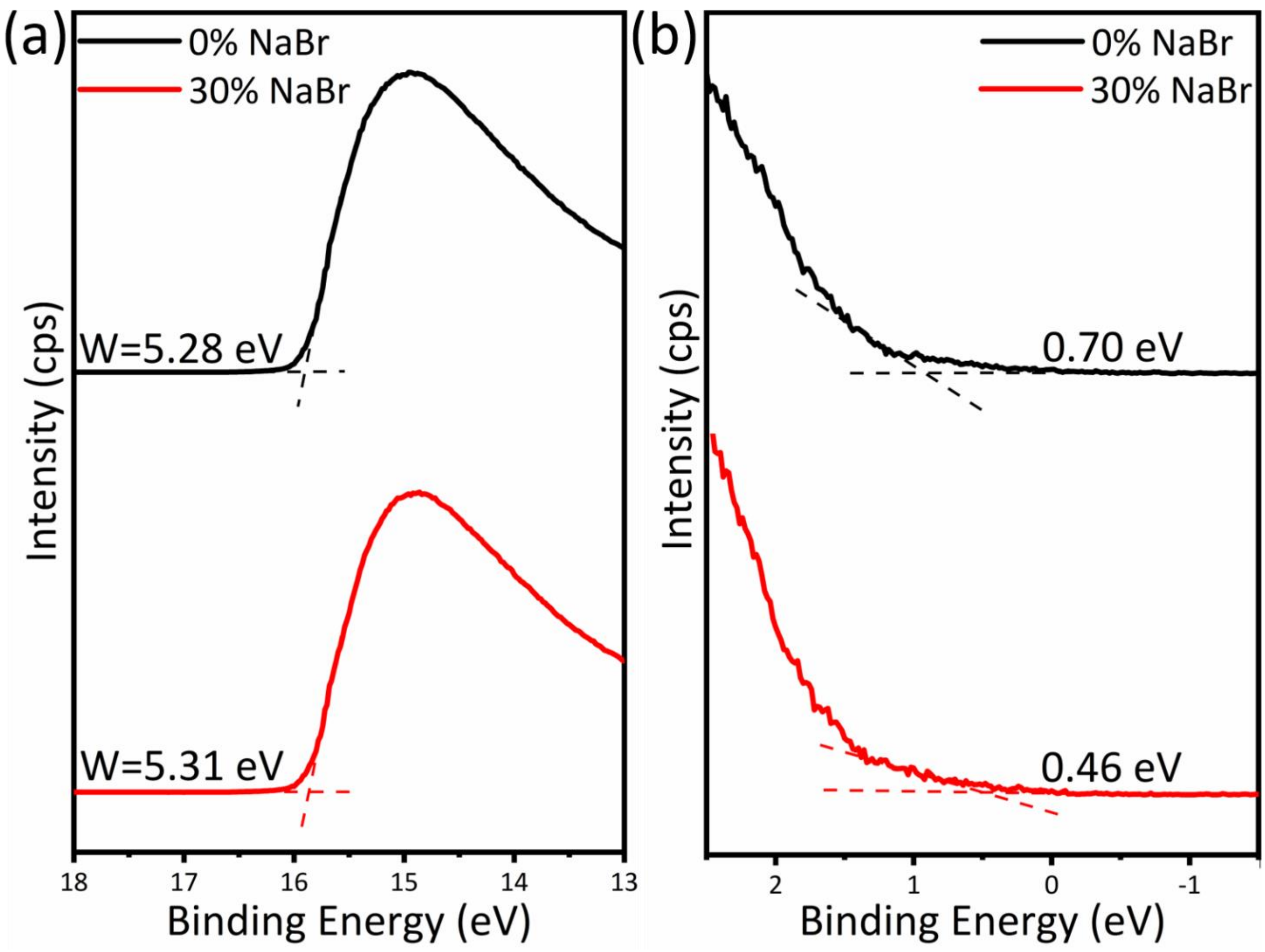

Figure S5. (a) High-binding energy secondary-electron cut-off regions of Q-2D perovskites without and with 30% NaBr. (b) VB-edge regions. The VB is calculated using the equation of VB = 21.2 – (E_{cutoff} – E_{onset}). The calculated VBs were 6.0 and 5.8 for 0% NaBr and 30% NaBr-based perovskite films, respectively.

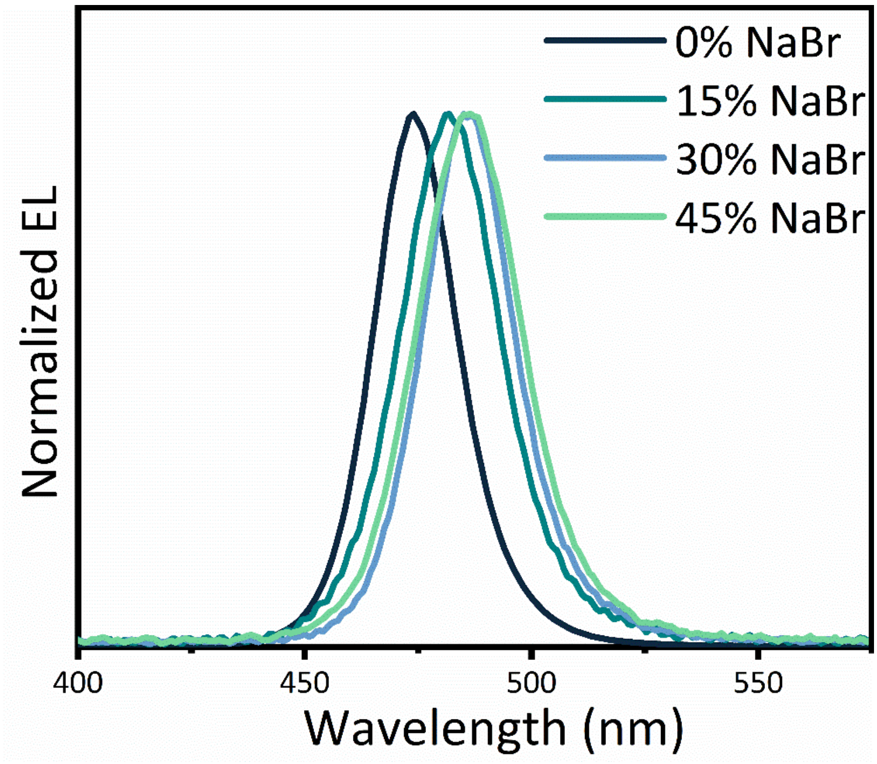

Figure S6. Electroluminescence (EL) spectra of perovskite LEDs.

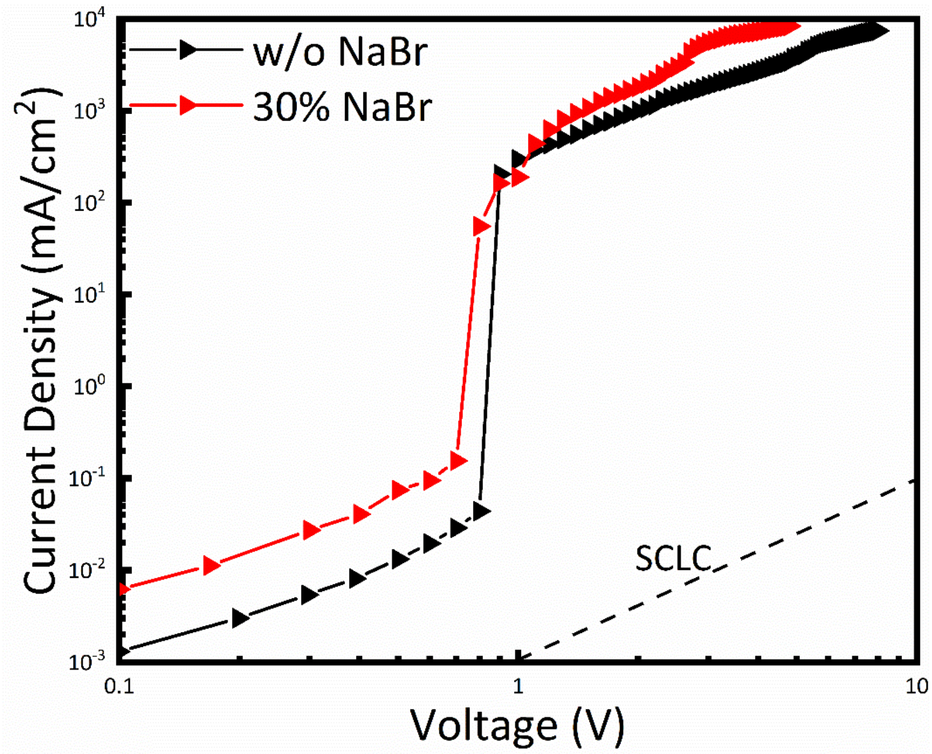

Figure S7. Hole mobility measurement of Q-2D perovskites without and with 30% NaBr by SCLC method, with a device structure of ITO/PEDOT:PSS/perovskite/MoO_3/Ag.

Table S2. Fitting parameters for the kinetics shown in **Figure 5e**. The kinetics are fitted by the multiple-exponential function, $\Delta A(t) = a_1 exp(-t/\tau_1) + a_2 exp(-t/\tau_2) + a_3 exp(-t/\tau_3) - c_1 exp(-t/\tau_{et})$, where a_1, a_2, a_3 and c_1 are the amplitudes; τ_1, τ_2 and τ_3 are the decay time constants and τ_{et} is the electron transfer time constant.

	**GSB [nm]**	τ_{et} **[ps]**	τ_1 **[ps]**	τ_2 **[ps]**	τ_3 **[ps]**	χ^2
n=2	414		5.1	45.6	8190.7	0.99
n=3	440		20.3	387.1	100000	0.97
n⩾4	475	30.4	290.5	20715.7		0.99

Table S3. Fitting parameters for the kinetics shown in **Figure 5f**. The kinetics are fitted by the multiple-exponential function, $\Delta A(t) = a_1 exp(-t/\tau_1) + a_2 exp(-t/\tau_2) + a_3 exp(-t/\tau_3) - c_1 exp(-t/\tau_{et})$, where a_1, a_2, a_3 and c_1 are the amplitudes; τ_1, τ_2 and τ_3 are the decay time constants and τ_{et} is the electron transfer time constant.

	**GSB [nm]**	τ_{et} **[ps]**	τ_1 **[ps]**	τ_2 **[ps]**	τ_3 **[ps]**	χ^2
n=2	418		4.8	43.0	8163.3	0.98
n=3	445		2.5	55.5	12707.5	0.99
n⩾4	488	22.2	229.4	16319.5		0.99

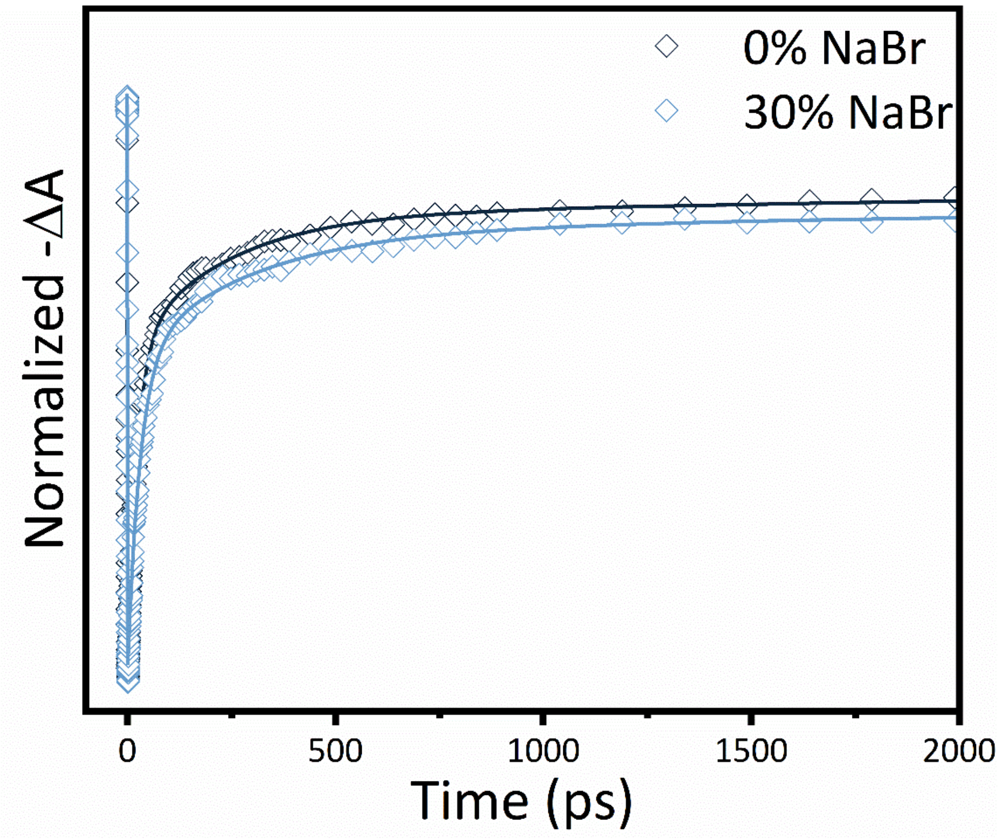

Figure S8. Bleach recovery dynamics for pristine and 30% NaBr-incorporated perovskite films. Solid lines are the fits of the kinetics by exponential function.

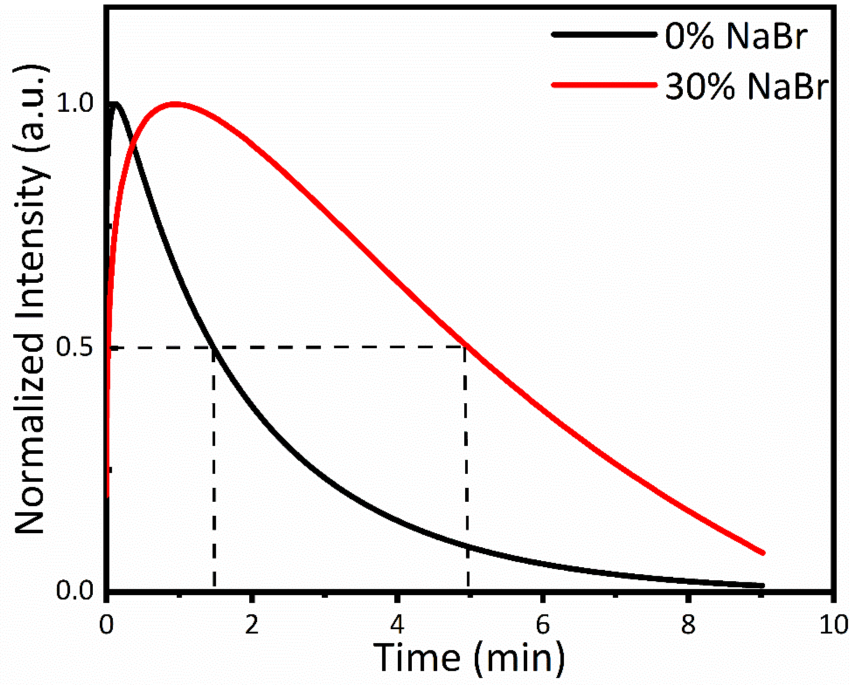

Figure S9. Stability measurement of the PeLEDs with an initial luminance of 100 cd/m^2.
